# Novel starter cultures *Virgibacillus* spp. selected from grasshopper sub shrimp paste to inhibit biogenic amines accumulation

**DOI:** 10.1186/s13568-021-01186-9

**Published:** 2021-02-10

**Authors:** Yirui Zhao, Xue Sang, Hongshun Hao, Jingran Bi, Gongliang Zhang, Hongman Hou

**Affiliations:** 1 School of Food Science and Technology, Dalian Polytechnic University, No.1, Qinggongyuan, Ganjingzi District, Dalian, 116034 China; 2 Liaoning Key Lab for Aquatic Processing Quality and Safety, Dalian Polytechnic University, No.1, Qinggongyuan, Ganjingzi District, Dalian, 116034 China

**Keywords:** Grasshopper sub shrimp paste, Starter cultures, Amine oxidases, Degradation of biogenic amines, Safety assessment

## Abstract

Controlling the content of biogenic amines (BAs) is critical to guarantee the safety of fermented aquatic products. The degradation characteristics and application potential of amine-negative starter cultures (*Virgibacillus halodenitrificans* CGMCC 1.18601: G25*, Virgibacillus pantothenticus* CGMCC 1.18602: G38) screened from grasshopper sub shrimp paste (Gssp) were studied. The enzymes of the two strains G25 and G38 that degrade BAs were amine oxidases (AOs) located on their respective cell membranes. The conditions that promoted the AO activity of *Virgibacillus* spp. were NaCl concentrations 5–10%, temperature 37 °C, pH 7.0 and ethanol concentrations 0–2%. Safety assessments (antibiotic susceptibility, biofilm activity and hemolytic activity) indicated that *Virgibacillus* spp. do not present a risk to human health, and this isolate can be confidently recommended as safe starter cultures for the food industry. Then, the two strains were cultured separately as starters and applied to the Gssp to analyze their influence on the flavor and quality of the product. As far as the bad flavors in Gssp such as sulfur-organic and sulf-chlor were concerned, the response values in the starter groups by G25 and G38 were significantly reduced by 39% and 65%, respectively. For the ability of strains to degrade BAs in Gssp, G25 degraded 11.1% of histamine, 11.3% of tyramine, 15.5% of putrescine and 4.1% of cadaverine; G38 significantly degraded 10.1% of histamine, 21.8% of tyramine, 18.1% of putrescine and 5.0% of cadaverine. These results indicated that the selected species could be used as starter cultures for the control of BA accumulation and degradation in Gssp.

## Introduction

For many years, fermented aquatic products have been very popular in Asia. They have a unique taste and their special flavor makes them a flavoring ingredient widely used in many cuisines. These products are highly salted, and fermentation normally takes several months (3–9 months), and the flesh may liquefy or form a paste during the process (Phewpan et al. 2019; Xinran et al. 2020). Some of these products include Nuoc-Mam (Nakano et al. 2018) of Vietnam and Cambodia, Nam-Pla (Natteewan et al. 2011) of Thailand, and Patis (Orejana and Liston, 2010) of the Philippines.

However, Asian fermented aquatic products pose a series of security risks. The most problematic safety issues are the presence of biogenic amines (BAs) (Spano et al. 2010; Kim et al. 2019). Poisoning from the ingestion of foods containing high levels of BAs has historically been referred to as scombroid poisoning because of the frequent association of this illness with the consumption of spoiled scombroid fish, such as mackerel and tuna. Additionally, anchovies, herrings, pilchards, sardines, shrimp, and some shellfish have been implicated in BA poisoning in Japan, Indonesia, and Sri Lanka, where food poisoning from aquatic products consumption is reported more often. Public health officials in many other countries acknowledge that BA poisoning occurs, but it is not officially required to be reported.

BAs are a group of low molecular weight organic compounds with biological activity and they contain nitrogen (Gardini et al. 2016). The most important BAs in fermented products are histamine (HIM), tyramine (TYR), putrescine (PUT) and cadaverine (CAD). They are mainly produced by decarboxylation of amino acids (Tan et al. 2019). Currently, there are many ways to control the content of BAs. The main measures are as follows: (1) reducing the level of amino acids in the precursor (Lorenzo et al. 2017); (2) inhibiting the growth of putrefactive microorganisms (Latorre Moratalla et al. 2007); and (3) using a strain that does not produce amino acid decarboxylase as a fermentation agent (Naila et al. 2010). When none of these methods can effectively reduce the BA content, the methods of adding microorganisms or BA-degrading enzymes can be used.

Many scholars have degraded BAs in food by inoculating microorganisms that do not have amino acid decarboxylase activity but have amine oxidase (AO) activity (Zaman et al. 2011). Degradation of BAs by AOs produced by some microorganisms is currently considered to be the most promising way to remove BAs from foods (Miguel et al. 2014; Callejón et al. 2014, 2016). Studies have shown that *Lactobacillus plantarum* ZY-40 can reduce the content of PUT and CAD in silver carp intestine by more than 70% (Zhang et al. 2013). *Staphylococcus epidermidis* R11 with AO activity in fermented sausage could help reduce HIM accumulation (Wang et al. 2015). *Lactobacillus plantarum* (ACBC271) and *Staphylococcus xylose* (CGMCC1.8382) were used as mixed fermentation agents to reduce the content of BAs in rice wine by 20.4% (Xiaole et al. 2018). *Lactobacillus curvatus* G-1 can degrade all BAs in Chinese bacon by more than 40% (Lu et al. 2018). Published researches gave evidence that *Vergibacillus halodenitrificans* can degrade histamine (Naila et al. 2012). Selecting proper starter cultures, optimizing fermentation conditions, and adding appropriate additives can help reduce BA formation and improve the quality of fermented products (Lee et al. 2012). Therefore, the screening of strains with AO activity as starters are of great significance for inhibiting the accumulation of BAs.

Grasshopper sub shrimp paste (Gssp) (Sang et al. 2020a) is a famous traditional fermented aquatic product in China. The grasshopper sub shrimps used in the production of the paste are mainly distributed around the Bohai Sea, where seawater and freshwater come into contact. Due to its unique flavor and excellent nutritional value, Gssp has been popular with a long history in China. However, it is traditionally manufactured by natural fermentation, which tends to lack quality and safety controls, and leads to the accumulation of BAs through microbial contamination.

In this study, we tried to isolate strains with biogenic amine (BA) degradation ability from grasshopper sub shrimp paste (Gssp) and defined it as non-BA producing. Among them, *Virgibacillus halodenitrificans* CGMCC 1.18601 (G25) and *Virgibacillus pantothenticus* CGMCC 1.18602 (G38) showing the strongest BA degradability were selected. Furthermore, the isolates were evaluated in terms of their potential as protective starter cultures based on a safety assessment. Then, we finally determined whether they can improve the bad flavors of the Gssp fermentation, and evaluated whether these strains are suitable for the preparation of Gssp.

## Materials and methods

### Samples and chemicals

BA standards, including histamine (HIM), tyramine (TYR), putrescine (PUT) and cadaverine (CAD), were purchased from Aladdin; acetonitrile and methanol were purchased from Spectrum Chemical; and L-histidine hydrochloride, L-tyrosine disodium salt, L-ornithine hydrochloride, and L-lysine hydrochloride were purchased from BBI. Acetonitrile and methanol were chromatographic grade, while all of the other chemicals were analytical grade.

### Strains and growth conditions

The research object came from 103 species kept in the laboratory, which were isolated from Gssp. The strains were grown in Luria–Bertani (LB) broth or on LB agar medium at 37 °C for 24 h. The strains were subcultured in LB broth for 24 h, and then streaked on LB agar medium, before being cultivated in LB broth for 24 h before use.

### Screening of strains

#### Decarboxylase activity

The decarboxylase activity of the strains was measured by culturing the strains in a liquid decarboxylase medium containing L-histidine hydrochloride, L-tyrosine disodium salt, L-ornithine hydrochloride and L-lysine hydrochloride. The color change of the medium at 48 h was monitored at 37 °C. Purple indicates a positive decarboxylase reaction, while yellow indicates it is negative (Eom et al. 2015).

#### AO activity

The AO test was carried out for the decarboxylase negative bacteria. The test method was as follows (Cheng et al. 2020): dip a white clean filter paper into the colony, add a drop of 1% dimethyl phenylenediamine hydrochloride solution, and then the solution turns pink and the color gradually deepens. After adding a drop of 1% α-naphthol ethanol solution, the positive color turned blue within half a minute, and the negative color was unchanged within two minutes. Positive cultures were the desired strains.

#### Evaluation of BA degradability

To evaluate the BA degrading activity of the amino acid decarboxylase negative and AO positive strains (Xu et al. 2016), the seed solution was inoculated into LB medium at a 1% inoculation volume, and then cultured at 37 °C and 150 r/min for 24 h. The cells were collected by centrifugation at 4000*g* for 10 min at 4 °C, washed twice with 0.05 mol/L pH 7.0 phosphate buffer solution, and then the bacteria were resuspended at four concentrations of 500 mg/L BAs (HIM, TYR, PUT and CAD) in 0.05 mol/L phosphate buffer with pH = 7.0, adjusting the bacterial suspension concentration to OD_600 nm=_0.8, and then cultured at 37 °C and 150 r/min for 48 h. The phosphate buffer solution containing BAs without any bacterial cells was kept under the same conditions for 48 h as a control group. After that, high-performance liquid chromatography (HPLC) was performed to quantify the BAs and to calculate the degradation rates of each BA by applying Formula 1.

1 $${\text{Degradation rates }}\left(\% \right) \, = \, ({\text{W}}_{0} \; - \;{\text{W}}_{{1}})/{\text{W}}_{0} *{1}00\%$$

Annotate: W_0_-The content of biogenic amines in the control group, mg/kg;

W_1_-The content of biogenic amines in the experimental group, mg/kg.

## Degradation of BAs under different conditions

### Effects of NaCl concentration on the BA degradation rates

The washed cells were resuspended in 0.05 mol/L phosphate buffer (pH 7.0) supplemented with 500 mg/L of each BA. The cell concentration was adjusted to OD_600 nm=_0.8. The NaCl concentration adjusted to achieve final concentrations of 0%, 5%, 10%, 15% and 20% (w/v). The mixtures were then incubated at 37 °C and 150 r/min for 48 h. Then, the BA degradation rates were determined.

### Effects of temperature on the BA degradation rates

The washed cells were resuspended in 0.05 mol/L phosphate buffer solution (pH 7.0, 10% NaCl concentration) supplemented with 500 mg/L of each BA. The cell concentration was adjusted to OD_600 nm=_0.8. The mixtures were then incubated at different temperatures (25, 30, 37, 40 and 45 °C) for 48 h with shaking at 150 r/min. The BA degradation rates were determined.

### Effects of pH on BA degradation rates

The washed cells were resuspended in sterile physiological saline with pH values of 5.0, 6.0, 7.0, 8.0, and 9.0, containing four BAs each at a concentration of 500 mg/L and salt concentration of 10%. We adjusted the concentration of the bacterial suspension to OD_600 nm=_0.8, and then incubated at 37 °C and 150 r/min for 48 h. The BA degradation rates were determined.

### Effects of ethanol concentration on the BA degradation rates

The washed cells were resuspended in 0.05 mol/L phosphate buffer (pH 7.0, 10% NaCl concentration) supplemented with 500 mg/L of each BA. The cell concentration was adjusted to OD_600 nm=_0.8. Ethanol was added to achieve final concentrations of 0%, 2%, 5%, 10% and 15% (w/v). The mixtures were then incubated at 37 °C and 150 r/min for 48 h. The BA degradation rates were determined.

### Degradation characteristics of biogenic amines

#### Ways of strain to degrade the BAs

The washed cells (Xu et al. 2016) were resuspended in 0.05 mol/L phosphate buffer solution (pH 7.0, 10% NaCl concentration) with an OD_600 nm=_0.8. A half volume of the suspension was used as the active cells group. The other half volume of the suspension was sterilized at 121 °C for 20 min and used as the dead cells group. BAs were added to both groups to achieve a final concentration of 500 mg/L of each BA. The mixtures were then incubated at 37 °C and 150 r/min for 48 h. Then, we could calculate the action mode of the strains to degrade BAs.

#### Localization of AOs

The culture was centrifuged at 8000 xg for 10 min (4 °C) and the supernatant was collected as the ‘extracellular fraction’. The cells were washed twice with 0.05 mol/L phosphate buffer (pH 7.0) and resuspended in phosphate buffer (pH 7.0, 10% NaCl concentration). The cell suspension was collected as the ‘whole cells’. We ground the cell suspension with a tissue grinder. The mixture was centrifuged at 10,000*g* for 30 min (4 °C) and the supernatant was collected as the ‘intracellular fraction’. The sediment was washed twice with 0.05 mol/L phosphate buffer (pH 7.0) and resuspended in 0.05 mol/L phosphate buffer (pH 7.0, 10% NaCl concentration), which referred to the ‘cell membrane fraction’. The BAs were added to the four groups to achieve a final concentration of 500 mg/L of each BA. All of the mixtures were incubated at 37 °C and 150 r/min for 48 h. The BA degradation rates of each group were determined (Tapingkae et al. 2010).

#### Safety assessment of the strains

The susceptibility of the strains to 8 clinically important antibiotics [chloramphenicol, clindamycin, erythromycin, ampicillin, kanamycin, streptomycin, tetracycline and ciprofloxacin: selected based on the EFSA guidelines for testing the antimicrobial susceptibility of the Bacillus species (EFSA 2012)] (Dina et al. 2020). The strains (Kanjan and Sakpetch 2020) were tested for hemolytic activity by streaking on blood agar (5% v/v sheep blood). The hemolytic reaction was detected by the appearance of a clear zone (β-hemolysis: complete lysis of red blood cells), a greenish zone (α-hemolysis: conversion of red blood cell hemoglobin to methemoglobin) or the absence of any zone (γ-hemolysis: no hemolytic activity) around the colony developing on the blood agar. The formation and measurement of biofilms mainly refers to previously reported experimental processes (Wang et al. 2012; Stepanovic 2000).

## Application analysis of BA-degrading strains

### Degradation of BAs in Gssp by strain

We added 500 µL of inoculum separately to a 100 mL Erlenmeyer flask containing 50 g of Gssp (Weihai, Shandong). The control sample was incubated with 500 µL of sterile distilled water instead of the starter. The mixture was then incubated at 37 °C for 10 days. The degradation rates of the BAs were determined by HPLC, and the experiment was conducted in triplicate.

### Effects of the strains on the flavor of the Gssp

We added 200 µL of inoculum separately to a 50 mL centrifuge tube containing a 20 mL Gssp sample obtained from Weihai, Shandong. Control samples were cultured with 200 µL sterile distilled water instead of the starter. Then, the mixture was incubated at 37 °C for 10 d. The PEN3 type electronic nose sensor was used to test the sample. Each chemical sensor corresponds to a different type of sensitive substance. The response signal of the electronic nose sensor is basically stable after 50 s. In this study, 58 s was selected as the signal acquisition time point, and each group of samples was measured 6 times in parallel.

### Degradation of BAs in other fermentation products by strains

Add 500 µL of inoculum separately to a 100 mL Erlenmeyer flask containing 50 g of cheese(SUKI,Germany) and natto(Japan) samples. The control sample was incubated with 500 µL of sterile distilled water instead of the starter. The mixture was then incubated at 37 °C for 10 days. The degradation rates of BAs were determined by HPLC, and the experiment was in triplicate.

### Determination of BAs

The contents of the BAs were determined by HPLC (Sang et al. 2020b). An aliquot (20 mL) of 10% trichloroacetic acid (TCA) was added to 5 g of the sample to be tested, and the mixture was homogenized using a vortex mixer and then allowed to react at 4 °C for 2 h. After that, the mixture was centrifuged at 3000*g* and 4 °C for 10 min. The supernatant was collected, whereas the residue was extracted again with an equal volume of 10% TCA and centrifuged as before. To measure 1 mL of bacterial solution, 9 mL of 10% TCA vortex was added, it was incubated at 4 °C for 2 h, then centrifuged at 3000*g* at 4 °C for 10 min. We mixed 400 µL of the abovementioned supernatant with 80 µL of 2 mol/L NaOH, 120 µL of saturated NaHCO_3_ and 800  µL of dansulfonyl chloride (10 mg/mL). The residual dansulfonyl chloride was removed by adding 50 µL of ammonia after 40 min in a water bath at 45 °C, followed by further incubation at room temperature for 30 min. This was followed by the addition of 550 L of acetonitrile, and it was centrifuged for 5 min at 4 °C and 3000*g*. The supernatant was filtered twice through a 0.22 µm filter before the determination of BAs.

## Statistical analysis

All statistical analyses were based on data from duplicate samples from experiments that were repeated three times. One-way analysis of variance (ANOVA) using SPSS 19.0 was used to determine whether differences between the strains were significant. The means were compared with Duncan’s multiple comparisons test, and p < 0.05 was considered to indicate statistical significance.

## Results

### Screening of BA-degrading strains in Gssp

Early in the laboratory, a total of 442 strains of halophilic bacteria were identified in the Gssp, and they were classified into 46 genera and 103 species based on their 16S rRNA sequences (Sang et al. 2020a). Inoculation amine-negative strains can reduce the accumulation of BAs from the source, and a decarboxylase negative result is indicated with a yellow color. As shown in the Additional file 1: Table S1, 37 strains were screened out.

**Fig. 1:**
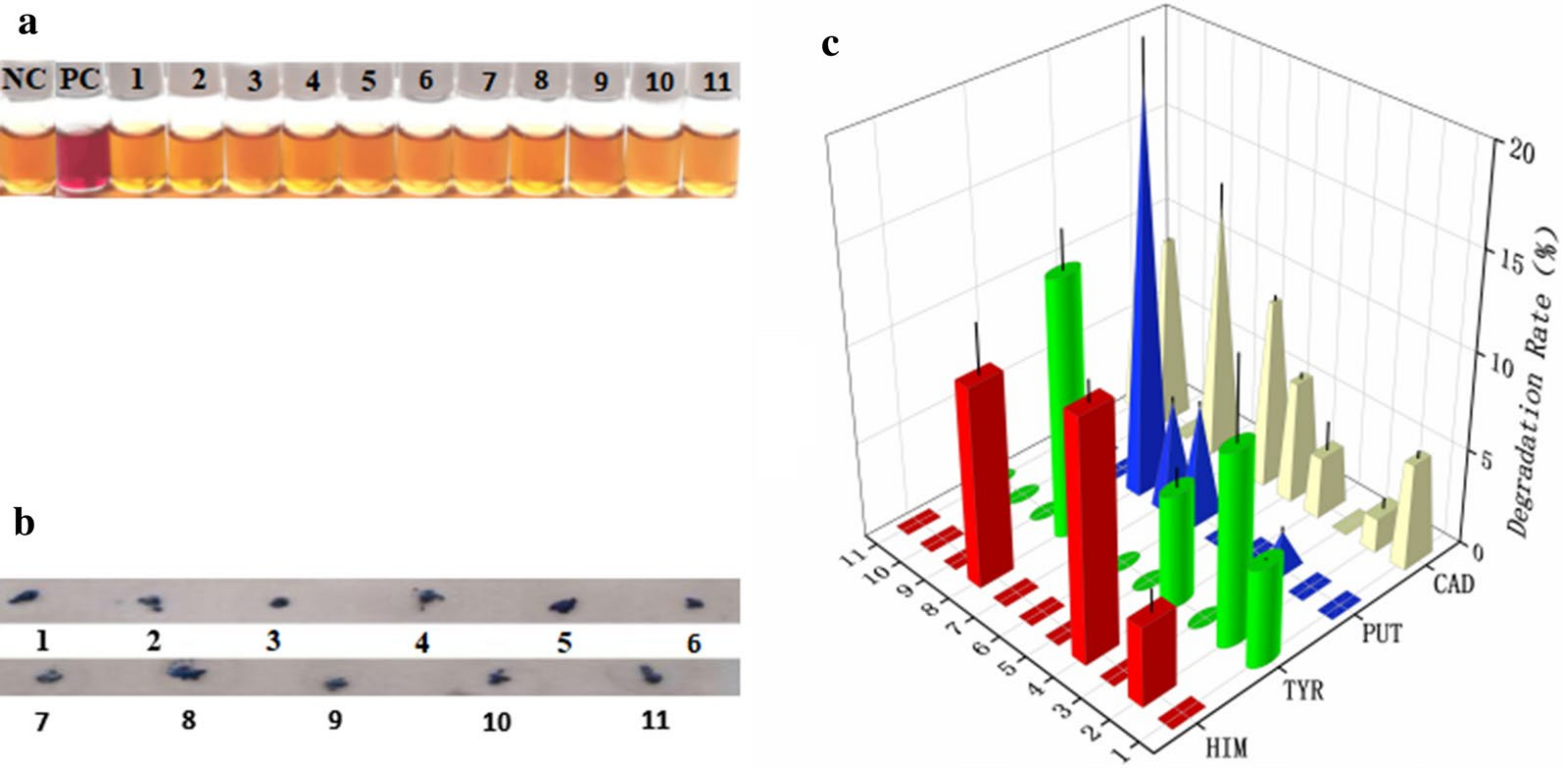
Screening of BA-degrading strains in Gssp (the numbers in the figure are the order of the strains): **a** Decarboxylase activity, negative reaction: yellow, the negative control (NC) is a medium without any bacterial cells; positive reaction: purple, the positive control (PC) strain is *Bacillus clausii*. **b** AO activity. **c** Evaluation of BA degradability, data are the means ± standard deviations from three determinations

AOs play a key role in the degradation process of BAs, and according to whether the colonies turned blue, 11 strains with strong AO ability were selected (Table 1, Fig. 1a, b). The degradation ability of these 11 strains is illustrated in Fig. 1c, and *Virgibacillus halodenitrificans* (G25) and *Virgibacillus pantothenticus* (G38) have the highest degradation rates among them. Therefore, follow-up studies were carried out on these two strains.

**Table 1:** Sequence number of BA degrading bacteria

Number	Strains
1	*Oceanobacillus picturae*
2	*Bacillus zhangzhouensis*
3	*Bacillus safensis*
4	*Virgibacillus halodenitrificans*
5	*Oceanobacillus oncorhynchi*
6	*Pantoea eucrina*
7	*Virgibacillus sp.*
8	*Virgibacillus pantothenticus*
9	*Bacillus cereus*
10	*Oceanobacillus caeni*
11	*Oceanobacillus iheyensis*

### Degradation of BAs under different conditions

**Fig. 2:**
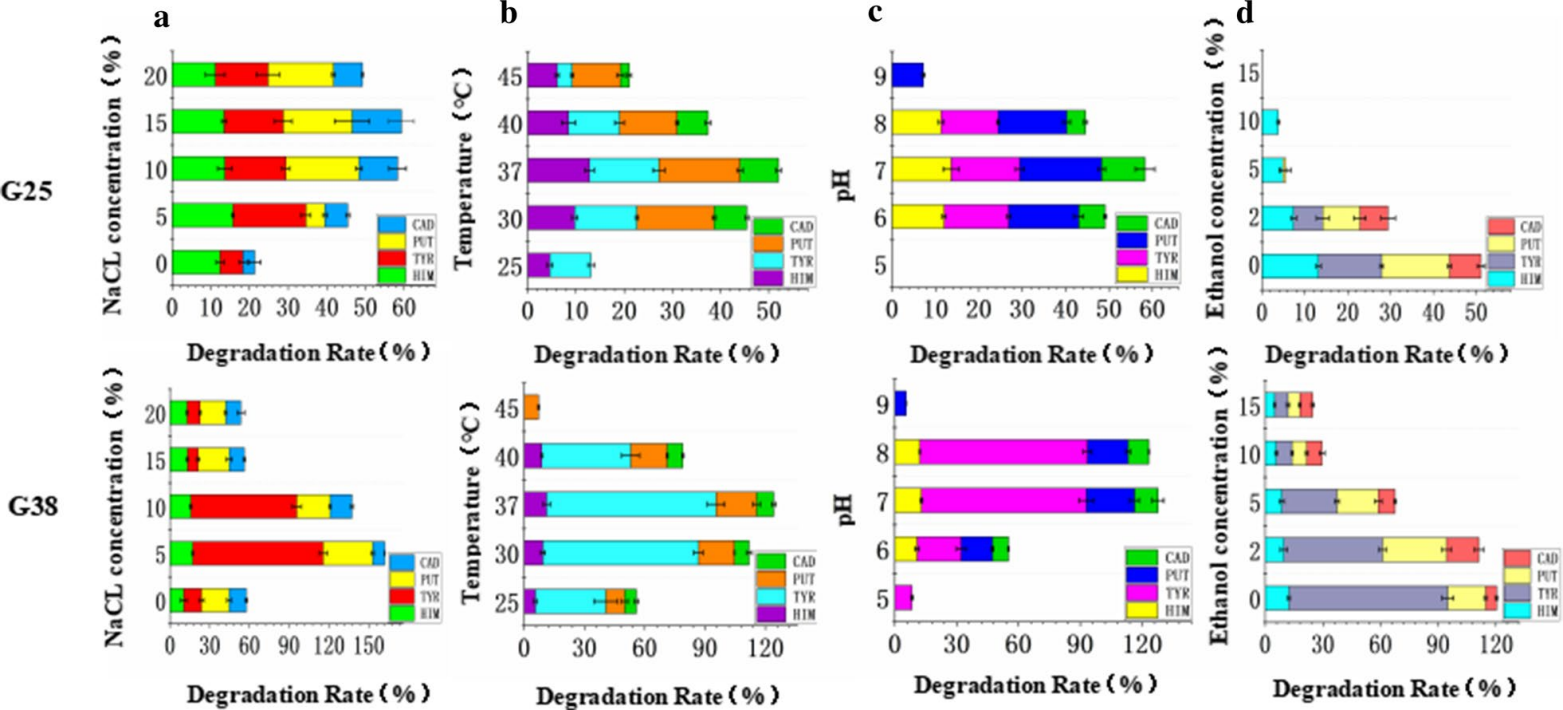
Degradation of BAs under different conditions, data are the means ± standard deviations from three determinations: **a** NaCl concentration. **b** Temperature. **c** pH. **d** Ethanol concentration

The degradation rates of the BAs reached the maximum, which also indicates that the activity of AOs is the strongest at this time. The effect of NaCl concentration is shown in Fig. 2a, and with an increase in NaCl concentration, the degradation rates of the BAs first increases and then decreases. The influence of temperature and pH on AO activity (Fig. 2b, c), they can be seen the best activity of AOs are at 37 °C and in the range of pH 6.0–8.0. The affect of ethanol concentration on the AOs is shown in Fig. 2d, the degradation rates of the BAs decreased rapidly with an increase in ethanol concentration, when the ethanol concentration reaches 10%, the degradation ability of most BAs is reduced by more than 50%.

### Degradation characteristics of biogenic amines

**Fig. 3:**
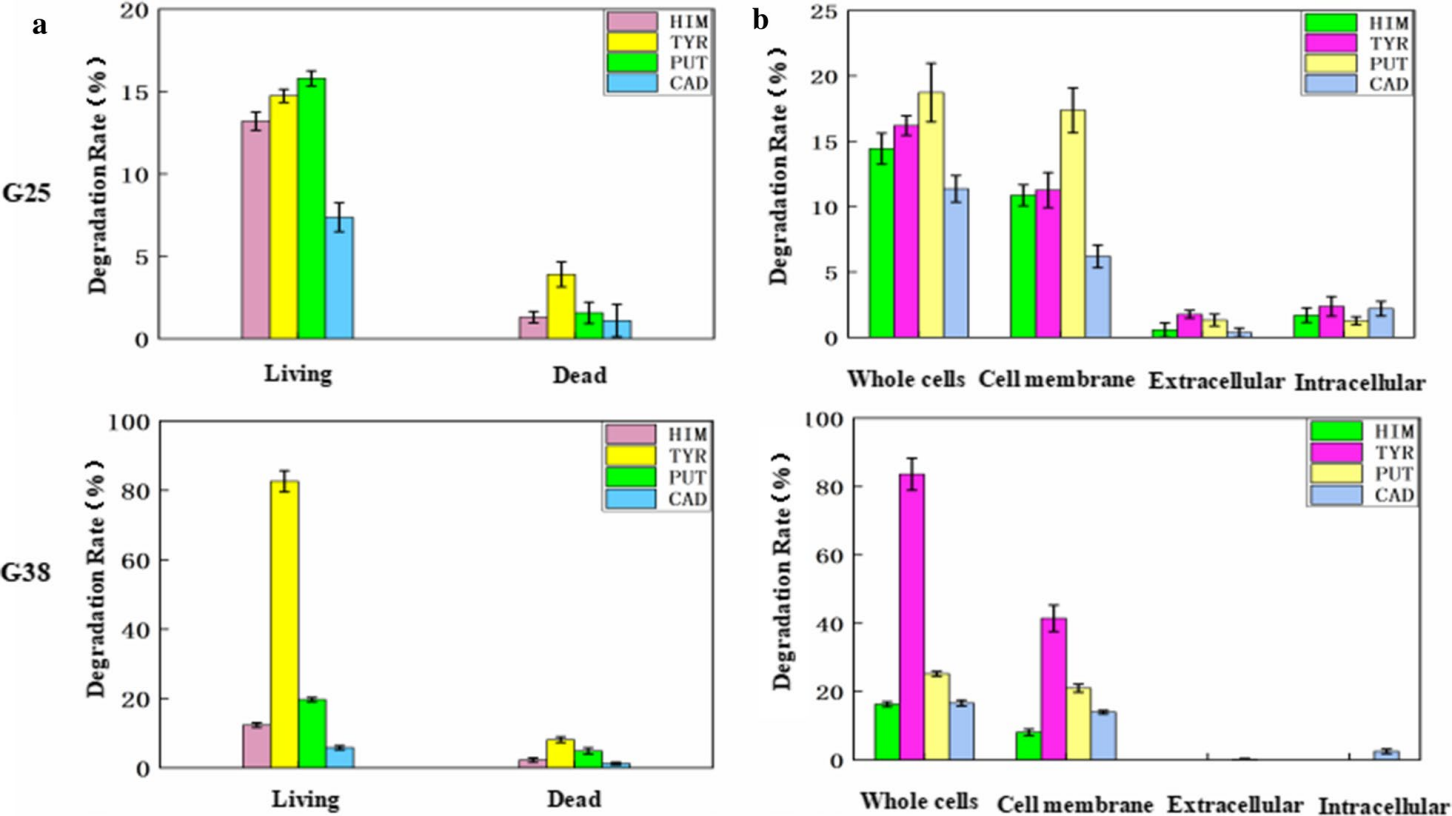
Degradation characteristics of biogenic amines, data are the means ± standard deviations from three determinations: **(a)** Ways the strains degrade BAs **(b)** Localization of the AOs

To determine whether the BA removal ability was due to adsorption or biological action, the removal rates of four BAs by dead cells and living cells of the strains were compared. The results are shown in Fig. 3a, the living cells showed high degradation ability on the four kinds of BAs, while the dead cells showed only very weak degradation ability on the four kinds of BAs. Thus, G25 and G38 removed four BAs mainly by their biological action.

Then, we determined where the AOs were located. It has been reported that AOs are mainly distributed on the cell membrane (Lizcano et al. 1998) or in the cytoplasmic space (Lee et al. 2008). As shown in Fig. 3b, BA degradation activity was not detected in either the extracellular or the intracellular fraction. The membrane fraction exhibited high degradation activity, although it was slightly lower than that of the whole cells. This might be attributed to partial AO destruction by broken tissue. Thus, it can be concluded that the AOs of G25 and G38 were mainly located on the cell membrane.

### Safety assessment of the strains

**Fig. 4:**
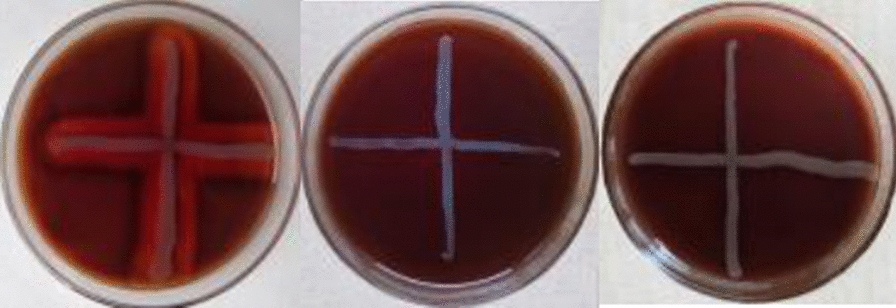
Hemolytic activity. From left to right, the positive control: *Bacillus Zhangzhouensis*, G25, G38

The safety attributes toward antibiotic susceptibility, biofilm activity and hemolytic activity were evaluated. As per EFSA (2012), the susceptibility of the strain to a certain range of antimicrobials of human and veterinary importance should be evaluated if the bacterial product needs to be used as an additive (Hae et al. 2017). It was found that these two strains were sensitive to the antibiotics analyzed and as per the MIC data, all of the values were lower than the breakpoint limit values as mentioned in EFSA (2012), as shown in Table 2. Biofilms are an organized and functional bacterial population. When bacteria form biofilms, their resistance to antibiotics will increase (Naicker 2016). As seen from Table 3, G25 and G38 showed negative results for biofilm formation ability. Hemolytic bacteria can easily cause hemolysis, leading to sepsis. We found G25 and G38 did not induce hemolysis when plated on sheep blood agar (γ-hemolysis), whereas *Bacillus zhangzhouensis*, as a positive control, induced β-hemolysis presenting with a clear zone around its colony (Fig. 4). Therefore, the combination of antibiotic susceptibility, biofilm activity and hemolytic activity test results can preliminarily determine that these strains are safe and have potential application prospects.

**Table 2:** Minimum inhibitory concentrations for eight antibiotics tested against the strains

Antibiotic	Minimum Inhibitory Concentration (mg/L)			
	MIC break points	Test sample		Interpretation
		G25	G38	
Chloramphenicol	8	8	8	s
Clindamycin	4	0.5	4	s
Erythromycin	4	0.5	4	s
Kanamycin	4	0.5	1	s
Streptomycin	8	1	2	s
Tetracycline	8	2	1	s
Ampicillin	4	0.5	0.5	s
Ciprofloxacin	4	0.5	4	s

**Table 3:** Biofilm activity of BA degrading bacteria

Control	Biofilm Determination		
	Test Sample		Activity
ODc	G25	G38	
0.04640	0.0061 ± 0.0017	0.01655 ± 0.0049	Negetive

### Application analysis of BA-degrading strains

**Fig. 5:**
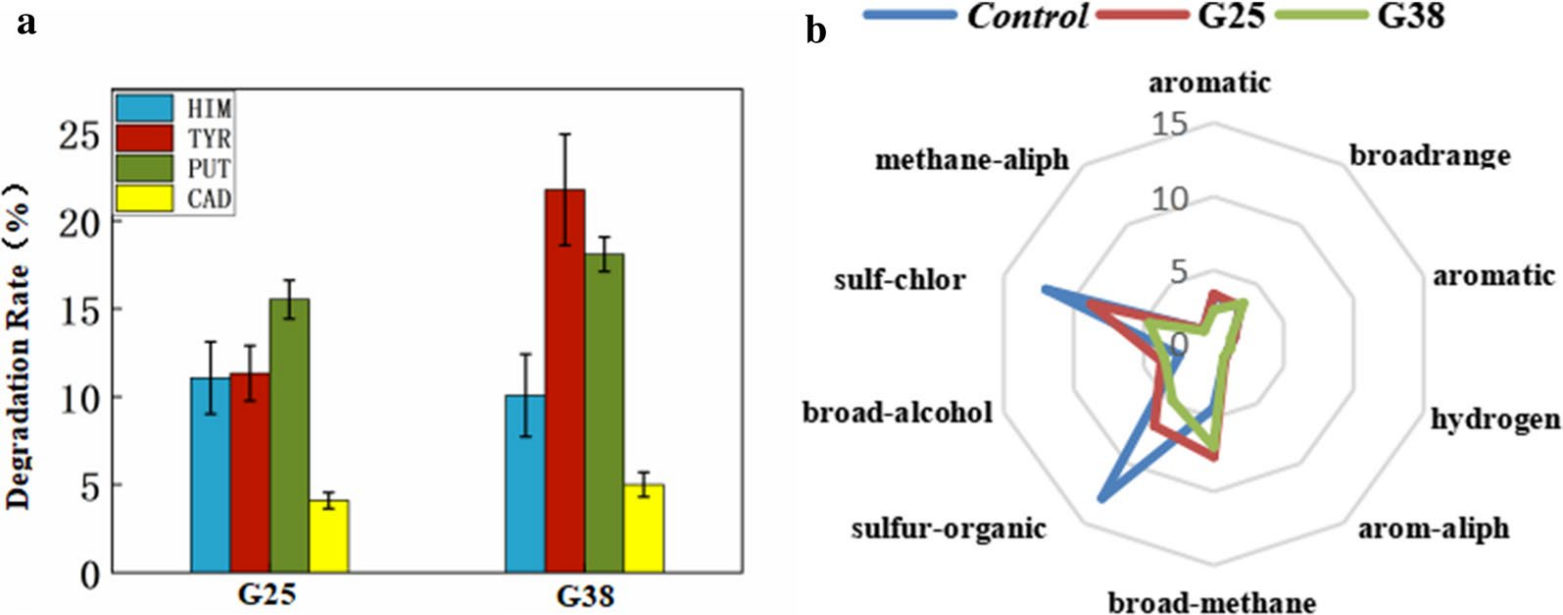
Application analysis of BA-degrading strains, data are the means ± standard deviations from three determinations: **a** Degradation of BAs in Gssp by strains. **b** Effects of strains on the flavor of Gssp

G25 and G38 might be potential candidates for controlling BAs due to their low BA-producing ability and high BA-degrading ability. Because the mixed culture of the strains has an unsatisfactory effect on the degradation of BAs (The results are not shown), the strains were cultured separately. To evaluate the BA degradation abilities of G25 and G38, we added 500 µL of inoculum separately to an Erlenmeyer flask containing 50 g of Gssp, then cultured it at 37 °C for 10 days. We determined the salt content and pH value of the Gssp sample to be 23.47% ± 1.68% (w/v) and 6.98 ± 0.14. The control sample without inoculation of strains showed the highest BA content (HIM: 17.72 ± 0.62 mg/kg, TYR: 26.30 ± 0.96 mg/kg, PUT: 52.60 ± 0.69 mg/kg, and CAD: 64.42 ± 0.22 mg/kg), which was due to fermentation of the native microbes in the fresh grasshopper sub shrimp. The degradation rates of the BAs were analyzed (Fig. 5a). G25 degraded 11.1% of HIM, 11.3% of TYR, 15.5% of PUT and 4.1% of CAD, while G38 significantly degraded 10.1% of HIM, 21.8% of TYR, 18.1% of PUT and 5.0% of CAD.

The dominant excellent flavor of Gssp is broad-methane, aromatic, broad-alcohol, etc. However, at the same time, the flavor substances of Gssp also contain some unfavorable substances, such as sulfur-organic and sulf-chlor, etc. When selecting starters, it is necessary to evaluate whether they have an adverse effect on the flavor or damage the taste of the products. We added 200 µL of inoculum (G25 and G38) separately to the centrifuge tube containing 20 mL Gssp and incubated it at 37 °C for 10 d. As seen from Fig. 5b, compared with the control, the broad-methane and broad-alcohol signal response value of the starter groups increased. For the sulfur-organic and sulf-chlor, the response values in the starter groups were significantly reduced, with G25 and G38 reduced by 39% and 65%, respectively.

**Fig. 6:**
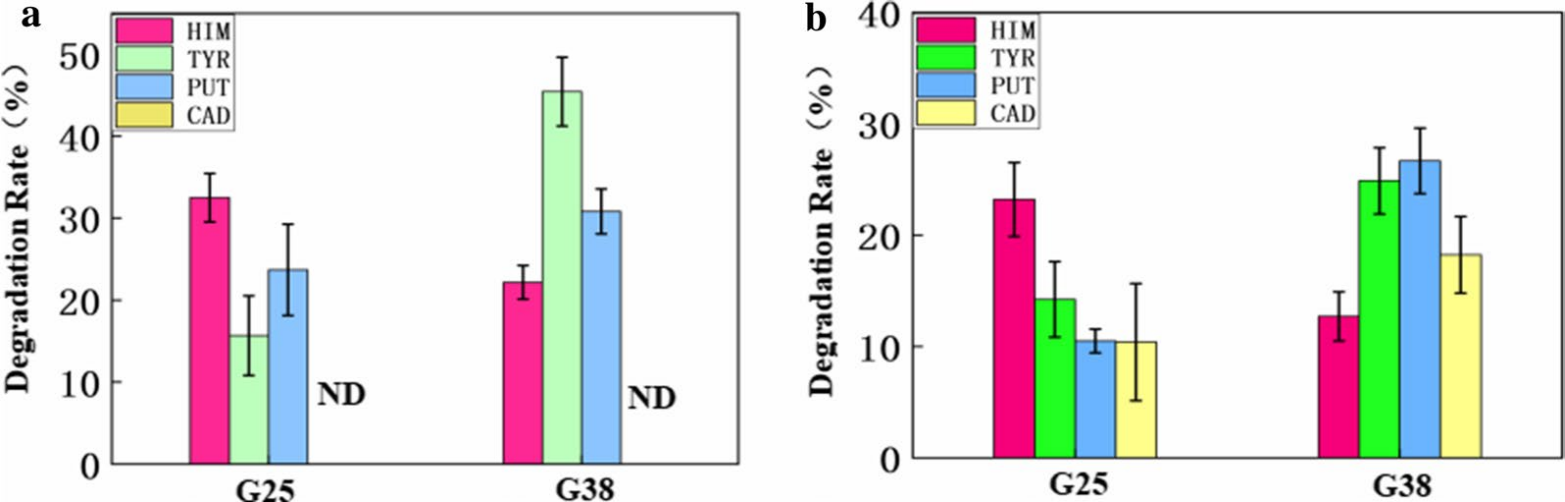
Degradation of BAs in other fermentation products by strains, data are the means ± standard deviations from three determinations: **a** Degradation of BAs in cheese by strains. **b** Degradation of BAs in natto by strains

In order to evaluate the degradation rates of BAs in other fermented products by strains, cheese and natto with higher BAs content were selected as the research objects. Evaluated the BAs degradation abilities of G25 and G38, add 500 µL of inoculum separately to Erlenmeyer flask containing 50 g of cheese or natto sample, after culture at 37 °C for 10 d. Determine the salt content and pH value of the cheese sample is 9.25% ± 0.87% (w/v) and 6.98 ± 0.14. For natto, sodium chloride is not added during processing and the pH value is 7.06 ± 0.27. The content of each BA in cheese are 17.82 ± 1.07 mg/kg of HIM, 51.30 ± 1.48 mg/kg of TYR, and 21.12 ± 1.81 mg/kg of PUT. The degradation rates of BAs in cheese were analyzed (Fig. 6a), G25 degraded 32.50% of HIM, 15.67% of TYR, and 23.70% of PUT; G38 significantly degraded 22.17% of HIM, 45.43% of TYR, and 30.83% of PUT. The content of each BA in natto are 17.16 ± 3.01 mg/kg of HIM, 37.52 ± 2.75 mg/kg of TYR, 17.62 ± 0.74 mg/kg of PUT, and 9.63 ± 0.57 mg/kg of CAD. The degradation rates of BAs in natto were analyzed (Fig. 6b), G25 degraded 23.20% of HIM, 14.23% of TYR, 10.50% of PUT, and 10.40% of CAD; G38 significantly degraded 12.70% of HIM, 24.87% of TYR, 26.67% of PUT, and 18.23% of CAD. In summary, these two starters still have high degradation rates for other fermentation products, they can also be used as starters in products other than Gssp.

## Discussion

*Virgibacillus* spp. are commonly found in fermented foods, but they appear to have no significant correlation with HIM, TYR, PUT or CAD, and it is the one of five most abundant genera of all bacteria in Gssp (Sang et al. 2020b). Many scholars have shown that the selected *Virgibacillus* spp. species could be used as starters for fermented aquatic products. For example, fish sauce samples inoculated with *Virgibacillus sp.* SK37 showed the potential to improve quality in terms of volatile compounds, glutamic acid content and overall acceptability (Udomsil et al. 2017). *Virgibacillus halodenitrificans* MSK-10P is a good candidate for further investigation for use in fermented shrimp paste to assess its technological performance as an autochthonous starter culture (Kumaunang et al. 2019).

G25 and G38 retained relatively high BAs degradation activity with an NaCl concentration ranging from 5 to 10% (Fig. 2a), this is because a certain amount of sodium chloride will change the permeability of the cell membrane and allow the AOs to penetrate more, when it exceeds a certain value, it will inhibit their permeability. For example, although SWA25 (Ying et al. 2016) is a salt-tolerant bacterium, the addition of NaCl concentration can significantly inhibit its BA degradation activity. *Virgibacillus* spp. had the highest degradation rate of BAs in the temperature at 37 °C (Fig. 2b), this may be because, on the one hand, a low temperature environment reduced the microbial activity, and on the other hand, the AO activity at high temperature is being strongly inhibited. This is consistent with the results of other relevant studies. For example, *halotolerant Staphylococcus carnosus* FS19 had the best ability to degrade HIM at 40 °C with a degradation rate of 23.5%, and when the temperature was increased to 45 °C and 50 °C, the degradation rates were only 6.1% and 3.3% (Zaman et al. 2014). *Brevibacillus sp.* SK35 had the highest HIM degradation rate at 35 °C (Sinsuwan et al. 2010). The highest BAs degrading activity were observed in the pH range of 6.0–8.0 (Fig. 2c), which may be because the strains degrade BAs mainly through AOs, and acid degradation plays a secondary role. At the pH values of 5.0 and 9.0, the degradation ability of the BAs are reduced or lost, which may be caused by the inhibition of the activity of microorganisms and AOs in an acidic or alkaline environment. Similar to this result, D05-1 had the highest HIM degradation ability (100%) at pH 7.0, but it slightly inhibited the degradation ability of HIM under acid or alkaline conditions (Lee et al. 2015). The inhibitory effect of ethanol on AOs (Fig. 2d), *Virgibacillus* spp. can only play a better role in the degradation of BAs in the ethanol system with a concentration of less than 2%, this result is consistent with previous research results that adding 12% ethanol reduced the degradation ability of *L. casei* IFI-CA 52 on HIM by approximately 70% (García Ruiz et al. 2011).

BA removal ability of G25 and G38 are mainly due to biodegradation, not adsorption or bioaccumulation (Fig. 3a) and AOs exist on the cell membrane (Fig. 3b). The activity of the AOs was affected by the NaCl concentration, temperature, pH and ethanol concentration. In particular, the concentration of NaCl can change the activity of AOs since at a suitable concentration, it can change the permeability of the cell membrane and increase the amount of AOs.

G25 and G38 can effectively remove BAs from Gssp (Fig. 5a), however, the complex environmental conditions of fermented foods, mainly high-salt environments, can inhibit the growth and reproduction of microorganisms while also reducing the activity of most AOs (Naila et al. 2012). *Virgibacillus* spp. can improve the flavor of Gssp (Fig. 5b), this is consistent with the findings of other scholars, Fonseca et al. (2013) proved that the overall acceptance of the final products inoculated with any of the starter cultures assayed was significantly higher than that of the uninoculated control batch.

In summary, *Virgibacillus* spp. species have potential uses as starters in a food model of fermented aquatic products. Two strains of G25 and G38 were found to be non-BA producing and actually degraded them. BAs are degraded mainly by AOs distributed on the cell membrane instead of by physical adsorption or bioaccumulation. Environmental factors have a strong impact on the activity of AOs. G25 and G38 can improve the flavor and quality of Gssp, in addition, these two strains were also found to be able to degrade the BAs in cheese and natto. G25 and G38 meet the relevant safety standards as starters, and may be used as effective biological control agents in fermented products to degrade the undesired accumulation of toxic BAs during product manufacturing. This research provides a certain theoretical basis for the selection and development of starters and the quality improvement of fermented products.

## Supplementary Information

**Additional file 1: Table S1.** 37 decarboxylase negative strains

## Data Availability

I would like to declare on behalf of my co-authors that the work described was original research that has not been published previously, and not under consideration for publication elsewhere, in whole or in part. All the authors listed have approved the manuscript that is enclosed.

## Abbreviations

BA: Biogenic amine
G25: *Virgibacillus halodenitrificans*
G38: *Virgibacillus pantothenticus*
Gssp: Grasshopper sub shrimp paste
AO: Amine oxidase
HIM: Histamine
TYR: Tyramine
PUT: Putrescine
CAD: Cadaverine
LB: Luria–Bertani
HPLC: High-performance liquid chromatography
