# Supplementary material for: Novel starter cultures Virgibacillus spp. selected from grasshopper sub shrimp paste to inhibit biogenic amines accumulation
Source: AMB Express. 2021 Feb 10;11:25. doi: 10.1186/s13568-021-01186-9 (PMC7876174; doi:10.1186/s13568-021-01186-9)
Supplement: Supplementary file 1 — Additional file 1: Table S1. 37 decarboxylase negative strains [file 13568_2021_1186_MOESM1_ESM.pdf]

Supplementary material

AMB Express

Novel starter cultures *Virgibacillus* spp. selected from grasshopper sub shrimp paste to inhibit biogenic amines accumulation

Yirui Zhao [1,2] . Xue Sang [1,2] . Hongshun Hao [2] . Jingran Bi [1,2] . Gongliang Zhang [1,2] . Hongman Hou [1,2*]

[1] School of Food Science and Technology, Dalian Polytechnic University, No.1, Qinggongyuan, Ganjingzi District, Dalian, 116034, China

[2] Liaoning Key Lab for Aquatic Processing Quality and Safety, Dalian Polytechnic University, No.1, Qinggongyuan, Ganjingzi District, Dalian, 116034, China

Address correspondence to Yirui Zhao, 1502701536@qq.com; No.1, Qinggongyuan, Ganjingzi District, Dalian 116034, China.

Address correspondence to Xue Sang, sangxue116@hotmail.com; No.1, Qinggongyuan, Ganjingzi District, Dalian 116034, China.

Address correspondence to Hongshun Hao, beike1952@163.com; No.1, Qinggongyuan, Ganjingzi District, Dalian 116034, China.

Address correspondence to Jingran Bi, 120256674@qq.com; No.1, Qinggongyuan, Ganjingzi District, Dalian 116034, China.

Address correspondence to Gongliang, Zhang, zhanggl1978@hotmail.com; No.1, Qinggongyuan, Ganjingzi District, Dalian 116034, China.

Address correspondence to Hongman Hou, houhongman@dlpu.edu.cn; No.1, Qinggongyuan, Ganjingzi District, Dalian 116034, China.

Tel.: +86-0411-8632-2020

Fax: 0086+ 0411—86322020

Decarboxylase negative strains

Additional file 1: Table S1. 37 decarboxylase negative strains

Number	Strains	Number	Strains
1	*Planococcus citreus*	20	*Cellulosimicrobium marinum*
2	*Paenibacillus lautus*	21	*Staphylococcus haemolyticus*
3	*Rhizobium pusense*	22	*Virgibacillus halodenitrificans*
4	*Dermacoccus barathri*	23	*Bacillus invictae*
5	*Allobacillus halotolerans*	24	*Bacillus zhangzhouensis*
6	*Pantoea eucrina*	25	*Sporosarcina saromensis*
7	*Staphylococcus xylosus*	26	*Virgibacillus pantothenticus*
8	*Sporosarcina koreensis*	27	*Psychrobacter sp.*
9	*Bacillus megaterium*	28	*Bacillus pacificus*
10	*Bacillus aryabhattai*	29	*Micrococcus yunnanensis*
11	*Staphylococcus epidermidis*	30	*Oceanobacillus picturae*
12	*Jeotgalicoccus halophilus*	31	*Staphylococcus cohnii*
13	*Oceanobacillus oncorhynchi*	32	*Tetragenococcus halophilus*
14	*Staphylococcus hominis*	33	*Bacillus cereus*
15	*Bacillus horikoshii*	34	*Halophilic Lactobacillus*
16	*Bacillus wiedmannii*	35	*Bacillus aerius*
17	*Bacillus safensis*	36	*Virgibacillus sp.*
18	*Bacillus pumilus*	37	*Oceanobacillus caeni*
19	*Oceanobacillus iheyensis*		
